# Role of Metabolism in Hepatic Stellate Cell Activation and Fibrogenesis

**DOI:** 10.3389/fcell.2018.00150

**Published:** 2018-11-12

**Authors:** Wei Hou, Wing-Kin Syn

**Affiliations:** ^1^Tianjin Second People’s Hospital and Tianjin Institute of Hepatology, Tianjin, China; ^2^Division of Gastroenterology and Hepatology, Department of Medicine, Medical University of South Carolina, Charleston, SC, United States; ^3^Section of Gastroenterology, Ralph H. Johnson Veterans Affairs Medical Center, Charleston, SC, United States

**Keywords:** fibroblast, glycolysis, glutaminolysis, liver fibrosis, metabolic

## Abstract

Activation of hepatic stellate cell (HSC) involves the transition from a quiescent to a proliferative, migratory, and fibrogenic phenotype (i.e., myofibroblast), which is characteristic of liver fibrogenesis. Multiple cellular and molecular signals which contribute to HSC activation have been identified. This review specially focuses on the metabolic changes which impact on HSC activation and fibrogenesis.

## Introduction

Activation of hepatic stellate cells (HSCs) involves the transition from a quiescent to a proliferative, migratory and fibrogenic phenotype (i.e., myofibroblast) which is characteristic of liver fibrogenesis. To date, multiple cell-surface, cytoplasmic and nuclear molecular signals and pathways have been reported to modulate HSC activation, including cytokines (Syn et al., 2011, 2012); adipocytokines (Saxena and Anania, 2015; Coombes et al., 2016); Toll-like receptors (TLRs) (Chou et al., 2012; Seo et al., 2016); Interleukins (ILs) (Jiao et al., 2016); collagen receptors (Liu et al., 2017); nuclear receptors (Beaven et al., 2011; Ding et al., 2013; Li et al., 2014; Palumbo-Zerr et al., 2015; Duran et al., 2016); G protein-coupled receptors (GPCRs) (Li et al., 2015, 2016a; Le et al., 2018); autophagy (Thoen et al., 2011, 2012; Hernández-Gea and Friedman, 2012; Hernández-Gea et al., 2012); endoplasmic reticulum stress (Hernández-Gea et al., 2013; Koo et al., 2016); oxidative stress (Lan et al., 2015; Ou et al., 2018); epigenetics (Coll et al., 2015; Hyun et al., 2016; Kweon et al., 2016; Huang et al., 2018; Zheng et al., 2018); cell metabolism (Nwosu et al., 2016; Du et al., 2018; Franko et al., 2018; Zhang et al., 2018), etc. In addition, extracellular/paracrine signals from resident and inflammatory cells including hepatocytes (Zhan et al., 2006), macrophages (Pradere et al., 2013), natural killer cells (Glässner et al., 2012), natural killer T cells (Wehr et al., 2013), liver sinusoidal endothelial cells (LSECs) (Xie et al., 2012), platelets (Kurokawa et al., 2016), and B cells (Thapa et al., 2015) further promote HSC activation.

In this review, we provide a focused update on the impact of cellular metabolism on HSC activation and fibrogenesis. A detailed discussion on other signals and pathways is beyond the scope of this article and has been reviewed elsewhere (Weiskirchen and Tacke, 2014; Lee et al., 2015; Wallace et al., 2015; Yang and Seki, 2015; El Taghdouini and van Grunsven, 2016; Hyun and Jung, 2016; Nwosu et al., 2016; Schumacher and Guo, 2016; de Oliveira da Silva et al., 2017; Higashi et al., 2017; Huang et al., 2017; Jiang et al., 2017; Kisseleva, 2017; Tsuchida and Friedman, 2017; Mortezaee, 2018; Ni et al., 2018; Wang J. N. et al., 2018).

## Aerobic Glycolysis: Warburg Effect

**FIGURE 1:**
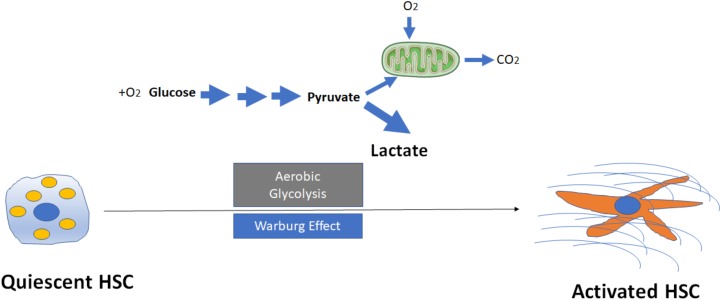
Activation of hepatic stellate cells (HSCs) through induction of aerobic glycolysis (Warburg effect). The transformation of glucose to lactate during HSC activation even when amounts of oxygen are available, leads to accumulation of intracellular lactate. Mitochondria may remain functional and some oxidative phosphorylation continue in cells. Aerobic glycolysis is less efficient than oxidative phosphorylation for generating adenosine 5′-triphosphate (ATP), which suggests that metabolites (for example, lactate) generated by aerobic glycolysis may have a more important role in the regulation of cellular functions than simply energy production during HSC activation.

Proliferative cells are often glycolytic, similar to the Warburg state that has been described in cancer cells. Diehl and colleagues first reported that reprogramming of quiescent hepatic stellate cell (Q-HSC) into myofibroblastic hepatic stellate cell (MF-HSC) is dependent upon induction of aerobic glycolysis (Chen et al., 2012). Compared with Q-HSC, MF-HSC express higher levels of glycolytic enzymes including hexokinase 2 (HK2), phosphofructokinase platelet (PFKP), pyruvate kinase M2 (PKM2) and glucose transporter 1 (GLUT1), monocarboxylate transporter 4 (MCT4), but downregulate key gluconeogenic enzymes phosphoenolpyruvate carboxykinase (PCK1) and fructose bisphosphatase (FBP1). During HSC activation, glycolysis occurs which lead to accumulation of intracellular lactate (Figure 1). Conversely, inhibition of conversion of pyruvate to lactate in MF-HSC with a pharmacologic inhibitor of lactate dehydrogenase A (LDHA) led to the decrease in lactate/pyruvate ratio, inhibition of proliferation, suppression of MF-genes expression, reduction of lipid accumulation and upregulation of genes involved in lipogenesis. Mechanistically, these investigators showed that activation of the Hedgehog (Hh) pathway upregulates expression of hypoxia inducible factor 1α (HIF1α), a key modulator of the expression and activity of glycolytic enzymes, directs glycolytic reprogramming, and controls the fate of HSC. By contrast, the inhibition of Hh signaling, HIF1α expression, glycolysis, or lactate accumulation results in the reversal of MF-HSC to a Q-HSC phenotype. These cellular changes are recapitulated *in vivo*: diseased livers of animals and patients accumulate an increasing number of glycolytic stromal cells that correlates with severity of liver fibrosis. In aggregate, these findings indicate that cellular metabolism plays a central role in the fibrogenic response, and imply that targeting cellular metabolism may be a novel antifibrotic strategy.

Despite these preliminary findings, the exact mechanisms that aerobic glycolysis modulates HSC activation and fibrogenesis remain largely unknown. For example, why (and how) do HSCs switch to aerobic glycolysis even when oxygen is abundant (Figure 1)? What are the key mediators to trigger the switch from oxidative phosphorylation to aerobic glycolysis? While glycolysis generates only two ATPs for each molecule of glucose, the oxidative phosphorylation produces up to 38 ATPs for each molecule of glucose that is consumed. Why should a cell utilize a less efficient metabolism system (at least in terms of ATP production) to promote HSC activation? Future studies will be needed to better understand the potential roles of lactate and lactate dehydrogenases (LDHs) in metabolic reprogramming. The current data to date, however, suggest that metabolites generated by aerobic glycolysis may have a more important role in the regulation of cellular functions then simply energy production.

## Glutaminolysis: Anapleurosis

**FIGURE 2:**
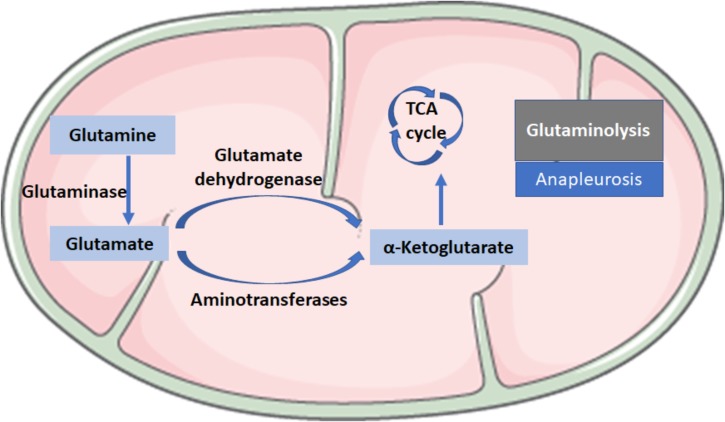
Biochemical reactions in glutaminolysis. Glutaminolysis is the conversion of glutamine (Gln) to α-ketoglutarate (α-KG) and consists of two reactions: the first reaction is catalyzed by the glutaminase (GLS), which converts Gln into glutamate (Glu) by losing an amino group; the second step consists of the conversion of Glu to α-KG, a critical intermediate in the tricarboxylic acid (TCA) cycle, which is catalyzed by glutamate dehydrogenase or aminotransferases.

Glutaminolysis is the conversion of glutamine (Gln) in α-ketoglutarate (α-KG) and consists of two reactions: the first reaction is catalyzed by the glutaminase (GLS), which converts Gln into glutamate (Glu) by loosing an amino group; the second step consists of the conversion of Glu to α-KG and is catalyzed by glutamate dehydrogenase or aminotransferases (Figure 2). Glutaminolysis could be involved in the mechanism for regulating HSC activation because glutaminolytic activity might fuel anapleurosis to meet the elevated demands of bioenergetic and biosynthetic pathways needed for the myofibroblastic phenotype.

In a recent study, Du et al. (2018) demonstrated that glutaminolysis could enable the transdifferentiation of HSCs into MF-HSCs. MF-HSCs, like highly proliferative cancer cells, are also highly dependent on glutamine *in vitro*. Glutamine is critical not only for MF-HSC growth but also for acquiring and maintaining a myofibroblastic phenotype. Their results show that α-ketoglutarate (α-KG), the end-product of glutaminolysis, helps to replenish the TCA cycle to satisfy the high bioenergetic and biosynthetic demands of MF-HSCs. Similar to aerobic glycolysis, investigators reported that Hh-mediated pathways also induce glutaminolysis to increase the production of energy and anabolic substrates needed to satisfy their increased demands when Q-HSC transdifferentiate to become MF-HSC. Interestingly, Yes-associated protein 1 (YAP) was identified as a downstream mediator of Hh-directed regulator of glutaminolytic enzymes during HSC transdifferentiation, and was shown to work in concert with its realted transcriptional regulator TAZ through TEAD binding sites to regulate glutaminase 1 (Gls1) expression in HSCs.

Similar findings were reported by Li et al. (2017). They showed that *culture*- as well as *in vivo*-activated HSCs demonstrate increased utilization of glutamine and related genes expression in glutamine metabolism, including glutaminase (GLS), aspartate transaminase (GOT1) and glutamate dehydrogenase (GLUD1). In addition to Hh signaling, TGF-β1, c-Myc, and Ras signaling have also been identified as major regulators of glutamine metabolism. In sum, these data indicate that increased glutamine metabolism not only meets an increasing energy demand but also functions as a key early regulator of HSC activation and fibrogenesis.

In support of its role in liver fibrogenesis, recent data also suggest that glutaminolysis regulates MF differentiation and play a critical role in other tissues. As an example, glutaminolysis was found to be a critical factor in the metabolic reprogramming of MF differentiation in lung tissues (Bernard et al., 2018), that TGF-β1 driven GLS1 expression is dependent upon both SMAD family member 3 (SMAD3) and mitogen activated protein kinase p38 (p38MAPK) activation.

Despite compelling data, further investigations are still needed to reveal the downstream components of the Hh-Yap-glutaminase axis, and identify alternative signaling pathways which regulate glutaminolysis in HSCs. It is also unclear if changes in glutaminolysis or other anaplerotic pathways, those catalyzed by pyruvate carboxylase (PC) as an example, can also modulate other regenerative programs and/or liver cells during fibrogenesis (Harvey and Chan, 2018).

## Lipid Droplets (LDs)

Q-HSCs are lipid-storing cells with the presence of large lipid droplets (LDs). During activation, HSCs lose their LDs (Friedman et al., 1993). LDs exist as a hydrophobic core of neutral lipids, surrounded by a phospholipid monolayer (Onal et al., 2017). In HSCs, the LDs contain in addition to neutral lipids consisting of triacylglycerols (TAG) and cholesterol esters, also retinyl esters (RE) with majority comprising of retinol/vitamin A. The exact mechanism of LD loss and its role in HSCs activation is unclear but has been recently studied. Cumulative data (Testerink et al., 2012; Tuohetahuntila et al., 2015, 2016, 2017; Ajat et al., 2017; Molenaar et al., 2017) show that LDs degrade during HSC activation in two distinct phases: (a) upon HSCs activation, the size of LDs was reduced while the number was increased during the first 7 days in culture; (b) disappearance of the LDs. During the prime stage of HSC activation there is a rapid decrease of REs, whereas the TAG content increases transiently, predominantly due to an abundant increase in polyunsaturated fatty acid (PUFA)-containing triacylglycerol, which is mediated by the increase in the ratio of the PUFA-specific fatty acid CoA synthase 4 (ACSL4) to the non-specific ASCLs, such as ASCL1.

**FIGURE 3:**
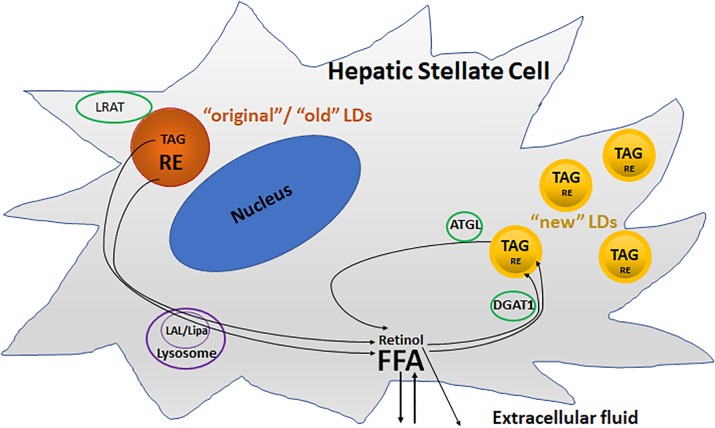
Two different metabolic pools of lipid droplets (LDs) in activated HSCs. The “original”/“old” LDs (depicted in brown), are located predominately round the nucleus, and contains predominantly triacylglycerol (TAG) and retinyl ester (RE), as well as retinol acyltransferase (LRAT). Lysosomal acid lipase (LAL/Lipa) is involved in the degradation of the “original”/“old” LDs in the lysosome during activation. The “new” LDs (depicted in yellow) which are smaller than “old” LDs, contain less REs but are enriched in TAGs, and are located in the periphery of the cells. Diacylglycerol O-acyltransferase 1 (DGAT1) and adipose triglyceride lipase (ATGL) are involved in the synthesis and breakdown of these newly synthesized TAGs, respectively.

Two pools of LDs are thought to exist in HSC: a preexisting (“original”/“old”) and a dynamic (“new”) pool of LDs (Molenaar et al., 2017; Tuohetahuntila et al., 2017; Figure 3). The preexisting LD pool, located predominately round the nucleus, containing predominantly TAGs and REs, as well as retinol acyltransferase (LRAT). During activation, lysosomal acid lipase (LAL/Lipa) is involved in the degradation of the preexisting LDs in the lysosome. The dynamic LDs, smaller than preexisting LDs, containing less REs but enriched in TAGs containing one or more PUFAs, are located in the periphery of the HSC. Diacylglycerol *O*-acyltransferase 1 (DGAT1) and adipose triglyceride lipase (ATGL), also known as patatin like phospholipase domain containing 2 (PNPLA2), are involved in the synthesis and breakdown of these newly synthesized TAGs, respectively.

Despite these new findings, the mechanism by which one pool is targeted for lipophagy and the other for lipolysis by ATGL remains elusive. It is also unclear why “old” types of LDs are degraded and “new” types of LDs formed. What triggers the replacement of retinyl esters by PUFAs? What kind of roles do the incorporated PUFAs play in contributing to HSC activation?

## Free Cholesterol (FC)

**FIGURE 4:**
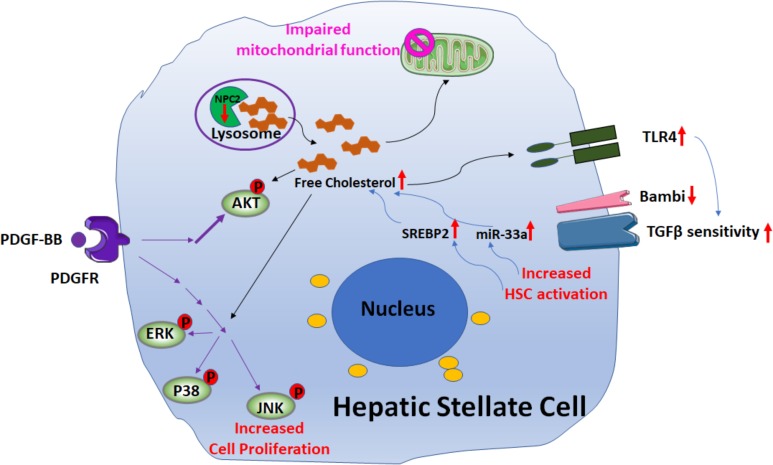
Signaling pathways involved in free cholesterol (FC) accumulation mediated HSC activation. Downregulation of Niemann–Pick type C2 protein (NPC2) results in FC accumulation and enhances platelet-derived growth factor BB (PDGF-BB)-induced HSC proliferation by extracellular signal-regulated kinases (ERKs), p38, c-Jun N-terminal kinases (JNK), and protein kinase B (AKT) phosphorylation. In addition, the mitochondrial respiration function is impaired. FC accumulation also increases Toll-like receptor 4 protein (TLR4) expression, thereby sensitizing cells to TGF-β-induced activation through down-regulation of TGFβ-pseudoreceptor Bambi. Along with HSC activation, subsequent upregulation of both sterol regulatory element-binding protein 2 (SREBP2) and miR-33a signaling leads to further FC accumulation and exaggerates liver fibrosis in a positive feedforward loop.

Recent studies (Schwabe and Maher, 2012; Teratani et al., 2012; Tomita et al., 2014a,b; Furuhashi et al., 2017; Figure 4) suggest that free cholesterol (FC) also mediates HSCs activation and fibrogenesis. FC accumulation in HSCs increases Toll-like receptor 4 protein (TLR4) levels by suppressing the endosomal-lysosomal degradation pathway of TLR4, and thereby sensitizes the cells to TGF-β-induced activation through down-regulating the expression of TGFβ-pseudoreceptor Bambi (bone morphogenetic protein and activing membrane-bound inhibitor). Along with HSC activation, subsequent upregulation of both sterol regulatory element-binding protein 2 (SREBP2) and miR-33a signaling through the suppression of PPARγ signaling, as well as disruption of the SREBP2-mediated cholesterol-feedback system in HSCs, which was characterized by a high SREBP cleavage-activating protein (Scap)-to- insulin-induced gene (Insig) ratio and exaggerated by the down-regulation of Insig-1 through the suppression of PPARc signaling, led to further FC accumulation and enhancing liver fibrosis in a positive feedforward loop. Notably, in a mouse model of liver fibrosis it was shown that reduction of FC accumulation in activated HSCs downregulated TLR4 signaling; this resulted in an increase of Bambi expression, which was associated with a reduction of liver fibrosis (Furuhashi et al., 2017).

Further support for the role of FC in liver fibrosis was demonstrated by studies on the Niemann–Pick type C2 protein (NPC2) (Twu et al., 2016; Wang Y. H. et al., 2018; Figure 4). NPC2 regulates intracellular cholesterol trafficking and homeostasis by directly binding with FC and expression of NPC2 is down-regulated in CCl4- and thioacetamide (TAA)-induced liver fibrosis tissues. The loss of NPC2 enhances the accumulation of FC in HSCs and exaggerates HSC response to TGF-β1 treatment. Gene depletion of NPC2 resulted in activation of extracellular signal-regulated kinases (ERKs), p38, c-Jun N-terminal kinases (JNK), and protein kinase B (AKT) phosphorylation which all contributed to increase the HSC proliferation induced by platelet-derived growth factor BB (PDGF-BB). In addition, the mitochondrial respiration function was also impaired.

Despite accumulating data on the role of FC on HSC phenotype, little is known of the roles of individual enzymes of cholesterol biosynthesis pathway in the fibrogenic response. Future studies will be needed to understand whether enzymes such as 3-hydroxy-3-methylglutaryl-CoA reductase (HMGCR), 3-hydroxy-3-methylglutaryl-CoA (HMG-CoA), mevalonate kinase (MVK), phosphomevalonate kinase (PMVK), diphosphomevalonate decarboxylase (MVD), farnesyl diphosphate synthase (FDPS), farnesyl-diphosphate farnesyltransferase 1 (FDFT1), squalene epoxidase (SQLE), 7-dehydrocholesterol reductase (DHCR7), or related metabolites may be involved in modulating HSC biology.

## Tricarboxylic Acid (TCA) Cycle

The tricarboxylic acid cycle (TCA cycle), also called Krebs cycle and citric acid cycle, which was proposed by Hans Adolf Krebs in 1937, is the final common pathway for oxidative catabolism of carbohydrates, fatty acids and amino acids, providing precursors for multiple biosynthetic pathways and plays a critical role in gluconeogenesis, transamination, deamination, and lipogenesis.

In brief, eight steps are involved in the TCA cycle, which is catalyzed by eight different enzymes including citrate synthase, aconitase, isocitrate dehydrogenase, ketoglutarate dehydrogenase, succinyl-CoA synthase, succinate dehydrogenase, fumarase, malate dehydrogenase. The TCA cycle starts with the convertion of the pyruvate into acetyl CoA, which is then converted in citrate by the combination with oxaloacetate. In a multi-steps reaction citrate is next converted in isocitrate to form then α-ketoglutarate. α-ketoglutarate loses a molecule of carbon dioxide and is oxidized to form succinyl CoA, which is then converted to succinate that is oxidized to form fumarate. At the end of the cycle fumarate is hydrolyzed to produce malate which is then oxidized to generate oxaloacetate. For each complete cycle there is the regeneration of oxaloacetate and the formation of two molecules of carbon dioxide.

**FIGURE 5:**
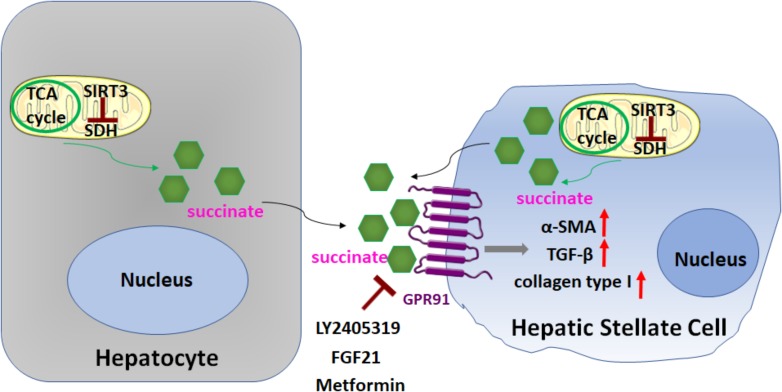
Role of succinate in HSC activation. Succinate, an intermediate in the TCA cycle, functions as a paracrine signal between hepatocytes and HSCs, through binding and activation of its cognate G protein-coupled receptor 91 (GPR91), which resulted in upregulation of fibrogenic markers alpha-smooth muscle actin (α-SMA), transforming growth factor β (TGF-β), and collagen type I. Sirtuin 3 (SIRT3), a NAD+-dependent protein deacetylase, predominantly localized in the mitochondrial matrix, is a key regulator of dehydrogenase (SDH) activity. The SIRT3-SDH-GPR91 axis regulates HSC activation. Repression of succinate-GPR91 signaling by LY2405319, an analog of fibroblast growth factor 21 (FGF21), as well as metformin inhibits HSC activation.

In a recent study Li et al. (2015, 2016a; Figure 5) demonstrated the importance of succinate (an intermediate in the TCA cycle) in HSC activation, through binding and activation of its cognate G protein-coupled receptor 91 (GPR91). When cultured HSCs were treated directly with succinate or with inhibitors of succinate dehydrogenase (SDH) (malonate, palmitate/choline, and methionine-choline deficient media), these resulted in the induction of GPR91, and upregulation of fibrogenic markers alpha-smooth muscle actin (α-SMA), transforming growth factor β (TGF-β), and collagen type I. Conversely, transfection of siRNA against GPR91 abrogated succinate-induced increases in the expression of α-SMA. Similar findings were observed when HSCs were isolated from methionine choline deficient diet-fed mice: HSC expressed higher levels of succinate, GPR91, and α-SMA. Taken together, these findings support a key role for succinate-GPR91 in HSC activation and fibrogenesis.

Sirtuin 3 (SIRT3), a NAD^+^-dependent protein deacetylase, predominantly localized in the mitochondrial matrix, is a key regulator of SDH activity. Recently, Li et al. (2016a; Figure 5) further found that the SIRT3-SDH-GPR91 axis regulated HSC activation, and proposed that succinate functions as a paracrine signal between hepatocytes and HSCs. Significantly, the repression of succinate-GPR91 signaling by LY2405319, an analog of the fibroblast growth factor 21 (FGF21), inhibited HSC activation. These observations suggest that the succinate-GPR91 pathway might be a potential therapeutic target in the treatment of liver fibrosis (Cho, 2018; Le et al., 2018; Figure 5).

## A Role for Direct Metabolism-Targeted Antifibrotic Strategy

Since both aerobic glycolysis (a target of the hedgehog pathway) and glutaminolysis (a process strongly regulated by Yap) are necessary to fulfill inherent metabolic requirements of the MF state and safely satisfies the bioenergetic and biosynthetic demands of highly proliferative cells, combining inhibitors of glycolysis and inhibitors of glutaminolysis which restrict both metabolic adaptations might be a physiologic and practical approaches to limit accumulation of MF-HSCs during liver injury.

Curcumin, a natural occurring principal curcuminoid of turmeric, has been reported to inhibit hedgehog signaling, decrease the accumulation of ATP and lactate, and downregulate the expressions and activities of hexokinase (HK) and phosphofructokinase-2 (PFK2) within HSCs. The glucose transporter Glut4 and lactate transporter MCT4 are also concomitantly downregulated (Lian et al., 2015). Thus, curcumin exhibits inhibitory effects on multiple steps of the glycolysis pathway and regulates metabolic reprogramming in activated HSCs (Lian et al., 2015), which is consistent with the report from Diehl and colleagues which showed that Hh signaling regulates metabolism in activated HSCs (Chen et al., 2012). In addition, as shown in a recent study (She et al., 2018), curcumin could also inhibit HSC activation via suppression of succinate-associated hypoxia-inducible transcription factor-1α (HIF-1α) induction.

Statins, are drugs known to lowering the levels of cholesterol and suppressing the cholesterol biosynthesis through the competitive inhibition of 3-hydroxy-3-methylglutaryl Co-enzyme A reductase (HMGCR) and subsequent blockade of the conversion of HMGCoA into mevalonate. Multiple studies have reported the potential antifibrotic roles of atorvastatin (Trebicka et al., 2010; Klein et al., 2012; El-Ashmawy et al., 2015; Ying et al., 2017), fluvastatin (Chong et al., 2015; Cheng et al., 2018), pitavastatin (Yang et al., 2010) and simvastatin (Wang et al., 2013; Jang et al., 2018), and recent data further reveal that these antifibrotic effects may occur via upregulation of the Krüppel-like factor 2 (KLF2) transcription factor (Marrone et al., 2013, 2015; Ray, 2015; Trebicka and Schierwagen, 2015).

Metformin, a well-known and the most widely used anti-diabetic drug, inhibiting hepatic gluconeogenesis in the liver, has been recently shown to suppress α-SMA expression via inhibition of succinate-GPR91 signaling in activated LX-2 cells (Nguyen et al., 2018; Figure 5). Interestingly, metformin can also attenuate activation of HSCs by activating the AMP-activated protein kinase (AMPK) pathway (Li et al., 2018; Nguyen et al., 2018). AMPK, recognized as an energy sensor with three heterotrimeric subunits (α, β, and γ), is an evolutionary conserved and ubiquitously expressed serine/threonine kinase playing a central role in the coordination of energy homeostasis. In a bleomycin model of lung fibrosis (Rangarajan et al., 2018), metformin therapeutically accelerates the resolution of well-established fibrosis in an AMPK-dependent manner through enhancing mitochondrial biogenesis and normalizing sensitivity to apoptosis. Metformin has emerged as novel antifibrotic strategies for the treatment of fibrotic diseases (Dos Santos et al., 2018; Li et al., 2018; Nguyen et al., 2018; Rangarajan et al., 2018).

## Conclusion and Speculation/Hypothesis

Cells constantly reprogram their metabolic pathways through direct or indirect mechanisms. Mounting evidences have shown the cross talk between signaling pathways and metabolic control in HSCs, and the complex interplay between metabolism and fibrogenesis is an exciting area of HSC research. Although recent data have shed light on the roles of some metabolic pathways in HSC biology, many more have yet to be described. A better understanding of the roles of cellular metabolism in HSC activation and fibrogenesis will provide us with novel molecular basis for the development of antifibrotic interventions.

Some speculative hypotheses might be put forward here to broaden the horizon about the role of metabolism in HSC activation and fibrogenesis. In addition to single cell glycolysis and glutaminolysis, symbiosis may be an alternate energy metabolism model that contributes to HSC fibrogenesis. For example, some HSCs might produce lactate with ATP production by consuming glucose (Warburg effect), while a neighboring HSC might consume the secreted lactate to produce ATP via the TCA cycle and oxidative phosphorylation. In fact, lactate could be used by some cancer cells [e.g., human non-small-cell lung cancers (NSCLCs)] as a substrate for TCA intermediates through monocarboxylate transporters (MCT1/4) and also for ATP production (Faubert et al., 2017).

Many glycolytic enzymes also function as protein kinases. Although these enzymes participate in specific metabolic pathways, each metabolic enzyme is also known to catalyze a unidirectional and/or bidirectional reaction. Recent data further revealed that pyruvate kinase M2 (PKM2), phosphoglycerate kinase 1 (PGK1), ketohexokinase (KHK) isoform A (KHK-A), hexokinase (HK), and nucleoside diphosphate kinases 1 and 2 (NME1/2) can function as protein kinases and phosphorylate multiple protein substrates to regulate cellular functions (Yang and Lu, 2015; Li et al., 2016b,c). Future studies will be needed to determine whether these, hitherto unrecognized protein kinase activity (of these metabolic enzymes) might also modulate HSC phenotype.

## Author Contributions

All authors listed have made a substantial, direct and intellectual contribution to the work, and approved it for publication.

## Conflict of Interest Statement

The authors declare that the research was conducted in the absence of any commercial or financial relationships that could be construed as a potential conflict of interest.
